# The face of control: Corrugator supercilii tracks aversive conflict signals in the service of adaptive cognitive control

**DOI:** 10.1111/psyp.13524

**Published:** 2020-01-13

**Authors:** Anja Berger, Vanessa Mitschke, David Dignath, Andreas Eder, Henk van Steenbergen

**Affiliations:** ^1^ Department of Psychology Universität Regensburg Regensburg Germany; ^2^ Department of Psychology Julius‐Maximilians‐Universität Würzburg Würzburg Germany; ^3^ Institute for Psychology Albert‐Ludwigs‐Universität Freiburg Freiburg im Breisgau Germany; ^4^ Leiden Institute for Brain and Cognition Leiden The Netherlands; ^5^ Leiden University Institute of Psychology Leiden The Netherlands

**Keywords:** conflict processing, EMG, emotion, motivation

## Abstract

Cognitive control is the ability to monitor, evaluate, and adapt behavior in the service of long‐term goals. Recent theories have proposed that the integral negative emotions elicited by conflict are critical for the adaptive adjustment of cognitive control. However, evidence for the negative valence of conflict in cognitive control tasks mainly comes from behavioral studies that interrupted trial sequences, making it difficult to directly test the link between conflict‐induced affect and subsequent increases in cognitive control. In the present study, we therefore use online measures of valence‐sensitive electromyography (EMG) of the facial corrugator (frowning) and zygomaticus (smiling) muscles while measuring the adaptive cognitive control in a Stroop‐like task. In line with the prediction that conflict is aversive, results showed that conflict relative to non‐conflict trials led to increased activity of the corrugator muscles after correct responses, both in a flanker task (Experiment 1) and in a prime‐probe task (Experiment 2). This conflict‐induced corrugator activity effect correlated marginally with conflict‐driven increases in cognitive control in the next trial in the confound‐minimalized task used in Experiment 2. However, in the absence of performance feedback (Experiment 3), no reliable effect of conflict was observed in the facial muscle activity despite robust behavioral conflict adaptation. Taken together, our results show that facial EMG can be used as an indirect index of the temporal dynamics of conflict‐induced aversive signals and/or effortful processes in particular when performance feedback is presented, providing important new insights into the dynamic affective nature of cognitive control.

## Abstract 2

Cognitive control plays a pivotal role in goal‐directed behavior. Nevertheless, it still remains elusive what mechanisms determine how cognitive control is recruited. Recent theories have proposed that negative emotions elicited by conflict help to adaptively increase the cognitive control. Although there is indeed accumulating evidence for the negative valence of conflict, no study has yet linked this directly to increased adaptive control. Using valence‐sensitive EMG measures, we here show that conflict is associated with increased activation of the corrugator (frowning) muscle and that the size of this effect predicts the size of conflict‐driven control adjustment in the next trial.

## 1 INTRODUCTION

Cognitive control is the ability to monitor, evaluate, and adapt our behavior in accordance with higher‐order goals and plans. This ability plays a pivotal role in daily life and has been shown to predict a wide range of outcomes including income, academic performance, and physical and mental health (de Ridder, Lensvelt‐Mulders, Finkenauer, Stok, & Baumeister, 2012). Nevertheless, it still remains elusive what mechanisms drive the adaptive recruitment of cognitive control. According to one influential theory, performance monitoring serves to inform and change cognitive control in an adaptive manner (Botvinick, Braver, Barch, Carter, & Cohen, 2001). To specify, this conflict monitory account has proposed that the conflict or incongruence between goal‐relevant and ‐irrelevant information in Stroop‐like tasks signals the need for additional cognitive control to prefrontal areas via the anterior cingulate cortex (Kerns et al., 2004). However, more recent work has suggested that conflict‐driven increases in cognitive control are not purely driven by cognitive processes but also involve affective processes (Braem, Verguts, Roggeman, & Notebaert, 2012; Dreisbach & Fischer, 2015; van Steenbergen, Band, & Hommel, 2009). Furthermore, activation patterns associated with cognitive control operations also overlap with the neural activation to pain (Kragel et al., 2018; Shackman et al., 2011), anxiety (Cavanagh & Shackman, 2015), and error monitoring (Moser, Moran, Schroder, Donnellan, & Yeung, 2013; Riesel, 2019). This work has inspired new theories proposing that negative emotions elicited by conflict trigger the subsequent increases in the recruitment of cognitive control, claiming that cognitive control depends on affective processes (Inzlicht, Bartholow, & Hirsh, 2015), and/or that the adaptation of control processes reflects an instantiation of affect regulation (Botvinick, 2007; Dignath, Eder, Steinhauser, & Kiesel, 2020; Dreisbach & Fischer, 2012a, 2015, 2016; Inzlicht et al., 2015; van Steenbergen, 2015).

To date, evidence for the negative valence of conflict in cognitive control tasks mainly comes from the behavioral studies showing that the conflicting Stroop stimuli are evaluated more negatively than non‐conflicting stimuli (Morsella, Gray, Krieger, & Bargh, 2009), facilitate categorization of negative stimuli relative to positive stimuli (Brouillet, Ferrier, Grosselin, & Brouillet, 2011; Dreisbach & Fischer, 2012b; Pan et al., 2016, Exp. 1) and lead to more negative evaluations of neutral stimuli (Damen, Strick, Taris, & Aarts, 2018; Fritz & Dreisbach, 2013, 2015; Regenberg, Häfner, & Semin, 2012, Exp. 1) and trigger motivational avoidance (Dignath & Eder, 2015). Conflict also modulates the reinforcement learning by acting as a signal of costs (Cavanagh, Masters, Bath, & Frank, 2014) and by providing a reward signal when solved (Schouppe et al., 2015, Exp. 1). Relatedly, inhibition of a dominant response tendency can also trigger stimulus devaluation (Wessel, O’Doherty, Berkebeile, Lindemann, & Aron, 2014), which corroborates a tight relationship between the evaluative and cognitive control processes. Furthermore, studies have shown that affective stimuli can modulate conflict adaptation, providing further evidence for a functional role of affect for control (Kuhbandner & Zehetleitner, 2011; Schuch & Koch, 2015; Schuch, Zweerings, Hirsch, & Koch, 2017; van Steenbergen, Band, & Hommel, 2009, 2010, 2015; but see Dignath, Janczyk, & Eder, 2017; Yamaguchi & Nishimura, 2019, Exp. 2 & 3). The functional link between the aversive quality of conflict and subsequent adaptation on the next trial has, however, only been investigated in paradigms where the original task was interrupted by inserting affective ratings in between trials (Fröber, Stürmer, Frömer, & Dreisbach, 2017). To examine the function of affective responses to conflict and subsequent behavior while not interrupting the task, we here will capitalize on the online recording of physiological measures that index participants’ affective state while they perform a typical conflict task.

Physiological measures in previous studies using Stroop‐like conflict tasks have already provided evidence that incongruent relative to congruent trials are accompanied by increased pupil dilation (Braem, Coenen, Bombeke, van Bochove, & Notebaert, 2015; D’Ascenzo, Iani, Guidotti, Laeng, & Rubichi, 2016; Diede & Bugg, 2017; Murphy, Van Moort, & Nieuwenhuis, 2016; van Steenbergen & Band, 2013; Wessel, Danielmeier, & Ullsperger, 2011), skin conductance response (Kobayashi, Yoshino, Takahashi, & Nomura, 2007), and increased heart‐rate slowing (Spapé & Ravaja, 2016; Spruit, Wilderjans, & van Steenbergen, 2018). The abovementioned measures are likely to reflect conflict‐modulated processes of attention and arousal rather than a hedonic or valence component. In the present study, we therefore use electromyography (EMG) measurements of the facial corrugator and zygomaticus muscles that produce frowning and smiling expressions, respectively.

Charles Darwin (1872) already noted that people of all cultures frown when they are puzzled, suggesting that effortful processes accompany frowning (see also Rinn, 1984; Shackman et al., 2011). The reduction of effort, by contrast, has been associated with smiling (Oster, 1978). These findings align with the notion that effort is intrinsically aversive (Kool, McGuire, Rosen, & Botvinick, 2010). Recording from the surface electrodes over the corrugator and zygomaticus muscles have been shown to be sensitive to cognitive effort (van Boxtel & Jessurun, 1993) and they also track the affective valence of participants’ affective state (Lang, Greenwald, Bradley, & Hamm, 1993), although this effect is more pronounced in the corrugator than in the zygomaticus (Larsen, Norris, & Cacioppo, 2003). Recent work has also shown that the facial EMG is sensitive to affective processes during cognitive tasks. In particular, it has been shown that the corrugator is reliably activated by errors (Elkins‐Brown, Saunders, He, & Inzlicht, 2017; Elkins‐Brown, Saunders, & Inzlicht, 2016; Lindström, Mattson‐Mårn, Golkar, Olsson, 2013; Dignath, Berger, Spruit, & van Steenbergen, 2019). The corrugator also responds to low levels of processing fluency, for example, if stimuli are difficult to process due to perceptual or conceptual features such as a low figure‐ground contrast, short presentation duration, or low semantic coherence (Cannon, Hayes, & Tipper, 2010; Forster, Leder, & Ansorge, 2016; Gerger, Leder, Tinio, & Schacht, 2011; Topolinski, Likowski, Weyers, & Strack, 2009, Winkielman & Cacioppo, 2001). One study indicated that the corrugator might be sensitive to response conflict, but this effect was only observed for a small subset of trials with very long reaction times (Lindström et al., 2013). An earlier study by Schacht, Dimigen, and Sommer (2010) reported a null finding in a Simon task. To the best of our knowledge, no study has found a modulation of corrugator and zygomaticus activation that would predict conflict‐driven adjustments in cognitive control.

The present research tested the idea that, if conflict is aversive and plays a functional role in cognitive control, it should (a) increase corrugator activity (and decrease zygomaticus activity) on incongruent relative to congruent trials, and (b) this effect should predict individuals’ behavioral conflict‐adaptation effect as indexed by the typical reduction of the congruency effect observed after incongruent versus congruent trials (Egner, 2007; Gratton, Coles, & Donchin, 1992).

## 2 EXPERIMENT 1

### 2.1 Method

#### 2.1.1 Participants

The Würzburg team (VM, DD, and AE) planned to collect data from *N* = 60 allowing them to detect correlations of *r* ≥ .4 between the behavior and physiology with a power of 80% and an alpha level of .05. Fifty‐nine students of the JMU Würzburg (aged 18 to 43, *M* = 25.29, *SD* = 4.89) participated in the experiment. Eleven of them were male and three participants reported to be left‐handed. All of them gave informed consent to participate and were remunerated for their participation after the experiment. One participant had to be excluded from behavioral analyses due to an extremely high error rate (25.08%) compared to the rest of the sample (*M*_sample_ = 5.62%, *SD* = 4.11). An additional 11 subjects were excluded from the fEMG data analyses due to recording errors or disturbances during the experiment. Finally, we screened the fEMG data for outliers separately for each cell of the factorial design (see below). No extreme outliers (i.e., more than three interquartile ranges below/above the 25th/75th percentile) were detected. The final sample for the fEMG analyses comprised *n* = 47 participants.

#### 2.1.2 Procedure

The participants’ skin was prepared for EMG measures before two (4 mm) electrodes above the areas of corrugator supercilii and zygomaticus major and one reference electrode were applied. EMG activity was amplified and recorded using a 16 channel V‐Amp system at 1,000 Hz (Brain Products, Gilching Germany).

The Flanker task was run using E‐Prime 2.0 software (Psychology Software Tools, Sharpsburg, PA, USA) on computers with 1,920 × 1,200 screens for stimulus presentation. Responses were collected using the D and L keys of a QWERTZ keyboard as left and right response buttons. Participants had to respond to flanker stimuli: Arrays of five letters consisting of H and S were presented; the middle letter served as the target stimulus and the flanking letters were distractors. The assignment of the response buttons to the target letters was balanced across subjects. Trials in which target and flanker letters corresponded (HHHHH, SSSSS) were congruent, trials in which they differed (HHSHH, SSHSS) were incongruent. There were 12 practice trials and 8 task blocks with 24 trials each. In each trial, a fixation sign was shown for 750 ms; the distractors without the target letter were presented for 100 ms; then the flanker stimulus was shown until registration of a response. Subjects received the performance feedback for incorrect responses (2,000 ms, “Falsch!”, German for “wrong!”) and for slow responses exceeding a time limit of 1,700 ms (“Zu langsam—reagiere schneller!”, “too slow—respond faster!”). The next trial started after an interval (ITI) of 2,000 ms.

#### 2.1.3 Data preprocessing

For error analyses, the first trial of each block (4.17%) was discarded. For RT analyses, trials with errors (5.36%), post‐error trials (4.86%), and all trials deviating more than 2.5 *SD* from the individual cell mean (2.02%) were additionally removed.

The EMG data were preprocessed with BrainVision Analyzer software (Brain Products Inc., Gilching, Germany). After filtering the data (20 Hz low cutoff filter, 500 Hz high cutoff filter, 50 Hz notch filter) we calculated the Root Mean Square (RMS) over 100‐ms time bins locked to the response. Artifacts were detected using a combination of methods (cf. Achaibou, Pourtois, Schwartz, & Vuilleumier, 2008; Lindström et al., 2013). Specifically, we removed outliers with (a) absolute activity for a given time‐bin and/or (b) its difference with the following time‐bin exceeding 3.5 *SD*s of its mean value. Mean and *SD* for these absolute and difference RMS values were calculated separately for each trial across time bins and across trials (for each bin separately). Artefacts were detected for each condition and participant separately in a time window from −300 ms to 1,500 ms relative to the registration of the participant's response. Data were segmented separately for the four different trial sequence conditions provided the response to that trial and to the preceding trial was accurate (congruent—congruent: cC, incongruent—congruent: iC, congruent—incongruent: cI, incongruent—incongruent: iI). The data were baseline corrected by subtracting the mean activity from 200 to 100 ms prior to the response from the activity in the rest of the bins (Elkins‐Brown et al., 2017). We analyzed the fEMG responses in the time window from response execution to 1,000 ms past response for 10 100‐ms time bins averaged across trials. Average EMG values were then *z*‐transformed for each participant and channel separately across the 10 time bins and four conditions. For reasons of completeness, analyses of the raw data (i.e., before *z*‐transformation) are reported in the Supporting Information.

#### 2.1.4 Design and analyses

As we were interested in conflict adaptation, both congruency of the current (congruency_N_, congruent or incongruent) and of the previous trial (congruency_N‐1_, congruent or incongruent) were within subjects‐factors in the behavioral analyses. A 2 × 2 repeated measures design was used to analyze the data for the dependent variables mean error rate (ER) and mean response time (RT). The dependent variable in the fEMG data were the standardized activation for a certain time bin (1–100, 101–200, …, 901–1,000 ms) as a function of congruency_N_ and congruency_N‐1_, resulting in a 2 × 2 × 10 repeated measures analysis of variance (ANOVA). ANOVAs were Greenhouse–Geisser corrected if necessary. In those cases the reported degrees of freedom were rounded. We also computed correlations of behavioral congruency effects (current incongruent minus current congruent; *I−C*) and CSEs (congruency effect after congruent minus congruency effect after incongruent trials; *[cI−cC]−[iI−iC]*) and physiological Flanker‐effects, that is, the fEMG responses, hypothesizing a positive correlation between these variables. We also report Bayesian *t* tests to interpret the null effects in Experiment 3. These tests were run using the JASP software package (version 0.10.2) using the Oosterwijk prior distribution (*t*‐distribution, centered at 0.35, with a scale of .102 and 3 *df*, Quintana & Williams, 2018) which is representative of the small‐to‐medium effects typically observed in psychological science (Gronau, Ly, & Wagenmakers, 2019).

### 2.2 Results

#### 2.2.1 Response times

The 2 × 2 ANOVA revealed a significant main effect of congruency_N_, *F*(1, 57) = 226.55, *p* ≤ .001, $\eta_{p}^{2}$ = .80, with faster responses in congruent trials (*M* = 389 ms, *SD* = 54 ms) compared to incongruent trials (*M* = 448 ms, *SD* = 66 ms). The effect of congruency_N‐1_ was also significant, *F*(1, 57) = 17.41, *p* ≤ .001, $\eta_{p}^{2}$ = .23. Responses were slower following incongruent trials (*M* = 422 ms, *SD* = 59 ms) relative to congruent (*M* = 415 ms, *SD* = 58 ms) trials. The interaction between both factors provided evidence for conflict adaptation [(*RT_cI_*−*RT_cC_*)–(*RT_iI_*−*RT_iC_*) = 18 ms; see Table 1], *F*(1, 57) = 16.32, *p* ≤ .001, $\eta_{p}^{2}$ = .22.

**Table 1:** Means, standard errors, and 95% confidence intervals of response times and error rates of all trial sequences and the respective congruency effects and conflict‐adaptation effects for each experiment

Measure		Experiment 1 (*n* = 58)			Experiment 2 (*n* = 27)			Experiment 3 (*n* = 38)		
		*M*	*SE*	95% CI	*M*	*SE*	95% CI	*M*	*SE*	95% CI
Reaction time (ms)	cC	381	7	[366, 395]	490	13	[463, 517]	475	10	[455, 495]
	cI	449	9	[431, 467]	581	15	[551, 611]	568	9	[550, 587]
	iC	397	7	[382, 412]	503	13	[476, 529]	488	10	[469, 508]
	iI	448	8	[431, 465]	573	14	[545, 601]	561	10	[541, 580]
	Conflict‐adaptation effect	18	4	[9, 25]	20	4	[12, 29]	21	3	[14, 28]
	Congruency effect	59	4	[52, 62]	81	5	[71, 90]	83	4	[75, 90]
	Overall	418	8	[403, 434]	537	13	[509, 564]	523	9	[504, 542]
Error rate (%)	cC	2.6	0.5	[1.7, 3.6]	5.2	0.8	[3.6, 6.9]	4.2	0.5	[3.1, 5.2]
	cI	8.7	0.9	[6.9, 10.4]	10.4	1.4	[7.6, 13.2]	10.9	1.0	[8.8, 12.9]
	iC	3.2	0.4	[2.3, 4.1]	5.0	0.9	[3.1, 6.8]	4.5	0.7	[3.2, 5.9]
	iI	6.6	0.7	[5.3, 8.0]	9.1	1.2	[6.6, 11.7]	9.2	1.0	[7.1, 11.3]
	Conflict‐adaptation effect	2.6	0.9	[0.2, 4.3]	0.9	0.6	[−0.3, 2.3]	2.0	0.8	[0.5, 3.5]
	Congruency effect	4.7	0.6	[3.2, 5.7]	4.7	0.8	[3.0, 6.3]	5.7	0.6	[4.4, 7.0]
	Overall	5.3	0.4	[4.4, 6.1]	7.4	1.0	[5.4, 9.5]	7.2	0.7	[5.7, 8.7]

Note

cC, cI, cI, and iI indicates the four possible sequences of congruent (c, C) and incongruent (i, I) trials with uppercase letters indicating current and lowercase letters indicating the previous trial type. The conflict‐adaptation effect was calculated as follows: (cI−cC)−(iI−iC). The congruency effect was calculated as follows: ((cI + iI)−(cC + iC)/2).

#### 2.2.2 Error rate

The ANOVA produced a main effect of congruency_N_, *F*(1, 57) = 55.55, *p* ≤ .001, $\eta_{p}^{2}$ = .49, with higher ERs in incongruent (*M* = 7.65%, *SD* = 4.91%) than in congruent trials (*M* = 2.92%, *SD* = 2.92%). The main effect of congruency_N‐1_ was *ns* , *F*(1, 57) = 2.264, *p* = .138, $\eta_{p}^{2}$ = .04. However, the interaction between congruency_N_ and congruency_N‐1_ reached significance, *F*(1, 57) = 7.43, *p* = .009, $\eta_{p}^{2}$ = .12, confirming adaptation to conflict [(*ER_cI_*−*ER_cC_*)–(*ER_iI_*−*ER_iC_*) = 2.58%; see Table 1].

#### 2.2.3 fEMG

**Figure 1:**
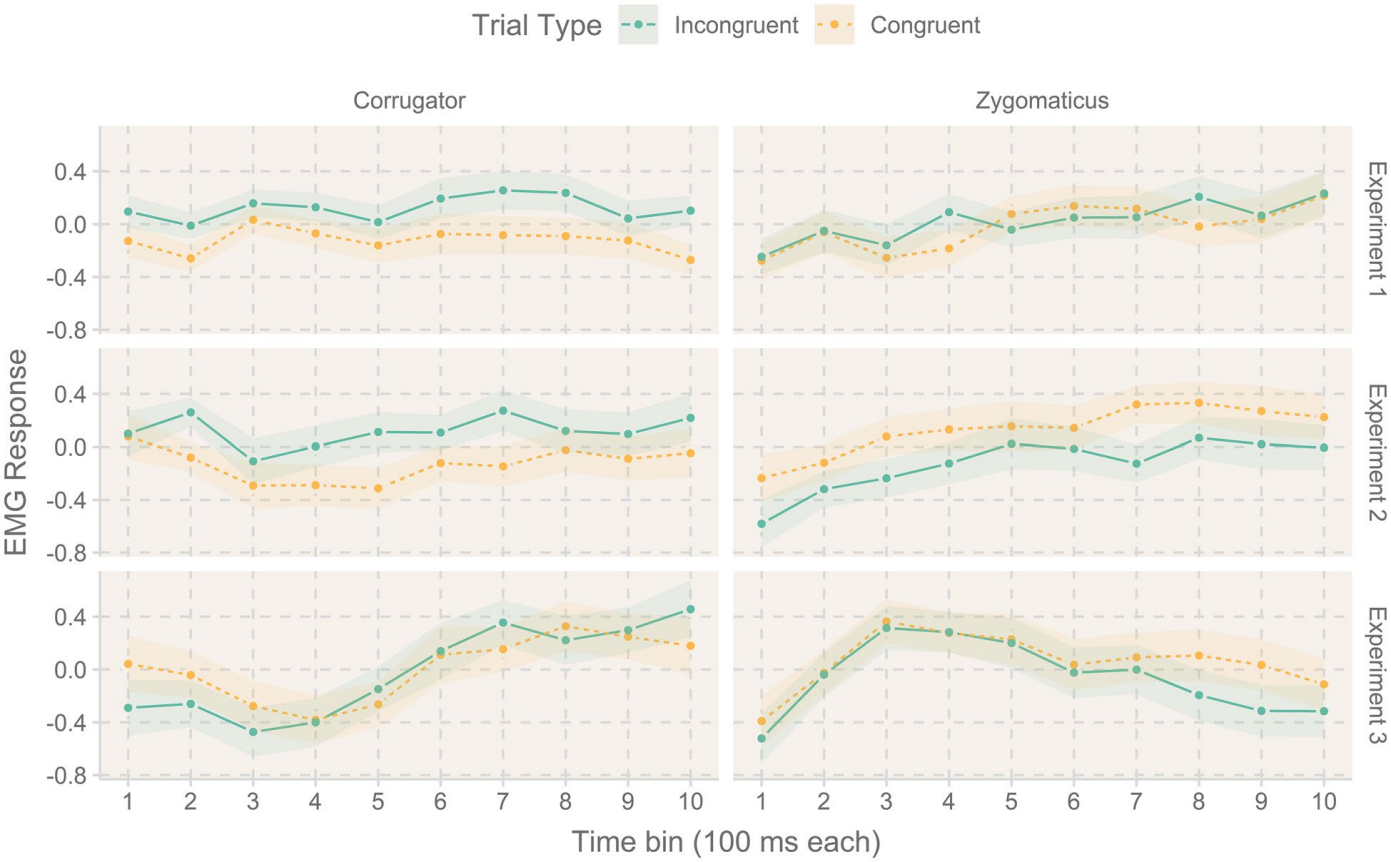
Grand averages of standardized EMG activation (RMS) of the corrugator and zygomaticus muscles as a function of each time bin and congruency in the three experiments. Shaded areas represent within‐subject standard errors of the grand means

Activation of corrugator supercilii was significant for congruency_N_, *F*(1, 47) = 7.73, *p* = .008, $\eta_{p}^{2}$ = .14, with stronger muscular activation in incongruent trials (*M* = 0.12; *SE* = 0.04; 95% CI [0.03; 0.21]) than in congruent trials (*M* = −0.12; *SE* = 0.04; 95% CI [−0.21; −0.03]; congruency effect: *M_CE_* = 0.24; *SE_CE_* = 0.09; CI [0.07; 0.42]). Figure 1 shows this congruency effect across the 10 investigated time bins. No other effects reached significance, all *F*s ≤ 0.78, all *p*s ≥ .577.

An analogous ANOVA for zygomaticus data revealed a significant effect of time bin, *F*(1, 9) = 4.64, *p* ≤ .001, $\eta_{p}^{2}$ = .09. Activation increased over time following a linear trend, *F*(1, 46) = 11.41, *p* ≤ .001, $\eta_{p}^{2}$ = .20. No other effects were significant, all *F*s ≤ 1.30, all *p*s ≥ .250.

The correlational analyses on the relationship between the fMEG congruency effect and behavioral CSEs did not produce significant results (−0.6 < *r*s < −.04, *ps* ≥ .686).

### 2.3 Discussion

Experiment 1 revealed increased corrugator activation in response to incongruent in comparison to congruent trials in a flanker task. The zygomaticus muscle did not show a reliable effect of congruency. Given that the corrugator muscle is responsive to negative emotions (Dimberg, 1990; Dimberg & Karlsson, 1997; Larsen et al., 2003), this finding provides the first evidence from a valence‐specific physiological measure that conflict during correct trials is aversive (Botvinick, 2007; Dreisbach & Fischer, 2016).

## 3 EXPERIMENT 2

The two‐choice flanker task used in Experiment 1 was not optimal because behavioral CSEs in this task could have been affected by the episodic memory processes related to the stimulus‐response repetitions and feature integration (Davelaar & Stevens, 2009; Hommel, 2004; Mayr, Awh, & Laurey, 2003; Nieuwenhuis et al., 2006). For example, Mayr et al. (2003) have proposed that the repetition priming during a Flanker task in which there are only two possible target stimuli and responses. All cC and iI sequences contain either complete stimulus repetitions (e.g., HHHHH to HHHHH) or complete switches (e.g., HHSHH to SSHSS), but none of the cI and iC sequences do so (i.e., HHSHH to SSSSS). This provides an explanation of CSEs in terms of the episodic memory rather than the adaptive control. Even when controlling for this confound, feature integration and contingency learning can still account for (part of) the CSE (for a review see Duthoo, Abrahamse, Braem, Boehler, & Notebaert, 2014).

Given these considerations, it is possible that processes other than the adaptive control masked a correlation between conflict‐induced corrugator activity and conflict adaption. In Experiment 2, we therefore used a prime‐probe task with four responses developed by Schmidt and Weissman (2014) that measures conflict adaptation without feature integration and contingency learning confounds.

### 3.1 Method

#### 3.1.1 Participants

The Leiden team (AB and HvS) planned to collect data from *N* = 30 for Experiment 2 and 3, respectively. The study was planned and conducted in parallel to and independently of the Würzburg team (Experiment 1). Sample sizes were large enough to detect medium‐to‐large effect sizes (*d_z_* ≥ 0.60) of conflict effects on the facial EMG with a power of 80% and an alpha level of .05. Thirty students of Leiden University aged from 18 to 27 years (*M* = 22.93, *SD* = 2.38) participated in exchange for 5€ or partial course credit after having signed the informed consent. All of them were right‐handed and five of them were male. Three subjects had to be excluded from behavioral analyses due to high (>2.5 *SD*) error rates (>28.64% vs. 8.5% sample mean). Two additional subjects had to be excluded from the fEMG analyses due to low EMG activation indicating a loose or broken electrode. Screening the remaining fEMG data for outliers separately for each cell (i.e., more than three interquartile ranges below/above the 25th/75th percentile) revealed one outlier. Final sample sizes were *n* = 27 for behavioral and *n* = 24 data sets for psychophysiological analyses.

#### 3.1.2 Stimuli and procedure

The participants’ skin was gently cleaned above the left corrugator supercilii (frowning muscle) and left zygomaticus major (smiling muscle) and on the forehead (ground signal) in order to prepare these areas for the fEMG signal recording. Five surface Ag/AgCl electrodes filled with electrode gel were applied to these regions. The EMG signal was acquired at 2,000 Hz using a BIOPAC MP150 combined with the EMG2‐R BioNomadix receiver. Stimulus and response onset markers were conveyed from the E‐Prime program via a parallel port and saved into an event marker channel. Data were stored using AcqKnowledge software (BIOPAC Systems Inc., Goleta, CA).

We used a modified version of the Stroop‐like conflict task developed by Schmidt and Weissman (2014). Each trial presented a blank screen (1,000 ms), a distractor (133 ms), a blank screen (33 ms), a target (133 ms), another blank screen (1,383 ms) during which the response was recorded, and a feedback screen (200 ms). The distractor consisted of three identical direction words (“Left,” “Right,” “Up,” or “Down”; 48‐point Courier New font) stacked vertically at the center of the display. The target was a single word at the center of the display (“Left,” “Right,” “Up,” or “Down”; 77‐point Courier New font). Participants were instructed to identify the target as quickly and as accurately as possible with pressing keys on a computer keyboard. More precisely, participants were to press F (left middle finger), G (left index finger), J (right middle finger), and N (right index finger) in response to “Left,” “Right,” “Up,” or “Down”, respectively. The word “Error” or “Too slow” (60‐point Courier new font) appeared as feedback after incorrect responses or no response, respectively. The task was presented on a 15‐inch monitor (1,280 × 1,024 px; 60 Hz) using E‐Prime version 2.0 software (Psychology Software Tools, Sharpsburg, PA, USA). All stimuli appeared in black on light gray background. Importantly, all odd‐numbered trials used a congruent or incongruent pairing of the words Left and Right while even‐numbered trials used a congruent or incongruent pairing of the words Up and Down. This procedure ruled out direct or indirect repetitions of particular stimuli and/or responses in two consecutive trials (Schmidt, 2013).

Participants performed a single block of 24 practice trials and subsequently eight blocks of 96 test trials (approximately 3 min each). Each block was followed by a self‐paced break.

#### 3.1.3 Data preprocessing

Like in Experiment 1, the first trial of each block (1.04%) was discarded for error analysis. For RT analyses, errors (10.05%), post‐error trials (8.05%), and all trials deviating more than 2.5 *SD* from the individual cell mean (2.11%) were additionally removed. The processing of fEMG data were done using the same methods as described in Experiment 1.

### 3.2 Results

#### 3.2.1 Response times

The 2 × 2 ANOVA revealed a significant effect of congruency_N_, *F*(1, 26) = 311.43, *p* ≤ .001, $\eta_{p}^{2}$ = .92, with higher RTs in incongruent (*M* = 577 ms, *SD* = 73 ms) than in congruent (*M* = 496 ms, *SD* = 67 ms) trials. The main effect of congruency_N‐1_ was* ns* , *F*(1, 26) = 1.04, *p* = .317, $\eta_{p}^{2}$ = .04. The interaction between congruency_N_ and congruency_N‐1_ was significant, *F*(1, 26) = 26.41, *p* ≤ .001, $\eta_{p}^{2}$ = .50, (*RT_cI_*−*RT_cC_*)–(*RT_iI_*−*RT_iC_*) = 20 ms (see Table 1).

#### 3.2.2 Error rates

The ANOVA produced a significant main effect of congruency_N_ (congruent: *M* = 5.09%, *SD* = 4.27%; incongruent: *M* = 9.76%; *SD* = 6.62%), *F*(1, 26) = 34.49, *p* ≤ .001, $\eta_{p}^{2}$ = .57, and a significant main effect of congruency_N‐1_ (congruent: *M* = 7.80%, *SD* = 5.27%; incongruent: *M* = 7.05%, *SD* = 5.18%), *F*(1, 26) = 6.60, *p* = .016, $\eta_{p}^{2}$ = .20. The interaction was *ns* , *F*(1, 26) = 2.47, *p* = .129, $\eta_{p}^{2}$ = .09.

#### 3.2.3 fEMG

The 2 × 2 × 10 ANOVA of corrugator activation revealed a main effect of congruency_N_,, *F*(1, 23) = 4.61, *p* = .043, $\eta_{p}^{2}$ = .18, indicating stronger activation in incongruent trials (*M* = 0.111; *SE* = 0.05; 95% CI [0.004; 0.218]) than in congruent trials (*M* = −0.111; *SE* = 0.05; 95% CI [−0.218; −0.004]); congruency effect: *M_CE_* = 0.22; *SE_CE_* = 0.10; CI [0.01; 0.44] see Figure 1). No other effects reached significance, *F*s ≤ 2.28, all *p*s ≥ .144.

Analyses of the zygomaticus activation showed a main effect of congruency_N_, *F*(1, 23) = 4.42, *p* = .047, $\eta_{p}^{2}$ = .16, with more activation in congruent (*M* = 0.13; *SE* = 0.06; 95% CI [0.002; 0.257]) than in incongruent trials (*M* = −0.13; *SE* = 0.06; 95% CI [−0.257; −0.002]; congruency effect: *M_CE_* = −0.259; *SE_CE_* = 0.123; CI [−0.515; −0.004]; see Figure 1). No other effects were significant, all *F*s ≤ 1.55, all *p*s ≥ .225.

**Figure 2:**
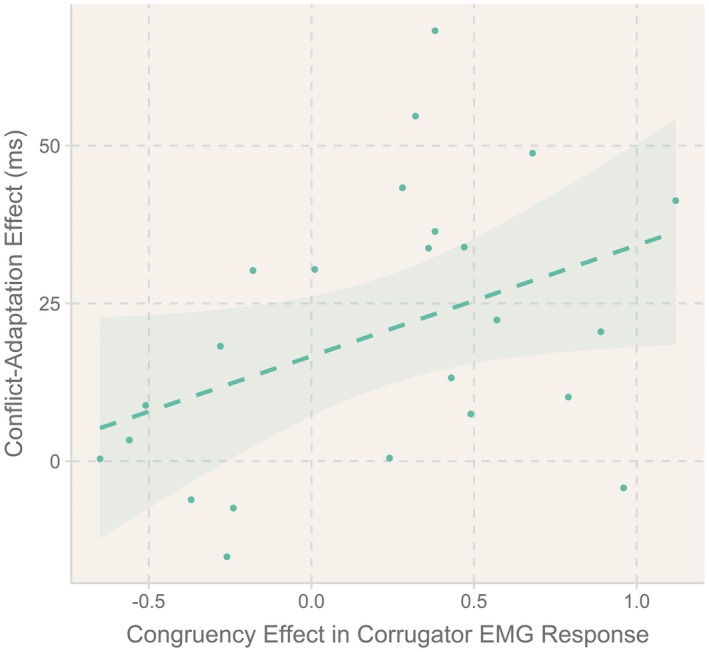
Positive correlation (*r* = .41) between the effect of conflict on the corrugator muscle (congruency effect) and behavioral conflict adaptation in reaction time

Correlational analyses revealed a marginally significant positive correlation of the congruency effect found in fEMG with the behavioral CSE of RT, *r*(22) = .41, *p* = .048 (see Figure 2), but no such correlation for the ER measure, *p* ≥ .52.

### 3.3 Discussion

Experiment 2 replicated a conflict‐induced increase in corrugator activation using a task in which SR congruency levels did not involve the systematic repetitions and/or changes of stimulus/response features. Moreover, it also showed that the strength of this signal in the corrugator marginally predicted the strength of behavioral conflict adaptation across the individuals (see Figure 2). This finding provides the first physiological evidence for a functional role of the averseness of conflict, corroborating earlier behavioral evidence using affective manipulations (Fröber et al., 2017; van Steenbergen et al., 2009). This experiment also revealed a reversed conflict effect in the zygomaticus major muscle, although we have to interpret this effect with caution because we did not observe this effect in Experiment 1 with a larger sample size.

## 4 EXPERIMENT 3

In Experiment 3 we aimed to examine more closely the processes underlying an enhanced corrugator activation in incongruent trials. Given that Experiment 1 and 2 used error feedback, and conflict trials generally lead to more errors than no‐conflict trials, the aversive response to error feedback could have become conditioned to incongruent trial displays, even when the participants responded correctly in these trials. Experiment 3 was the same task as Experiment 2 with the exception that the error feedback in between trials was replaced by a fixation cross.

### 4.1 Method

#### 4.1.1 Participants

Thirty‐eight students of Leiden University (three male), aged from 18 to 30 years (*M* = 22.50, *SD* = 2.82) participated for monetary compensation or partial course credit.1While collecting data we identified that some data were not recorded correctly due to a broken electrode. We therefore collected data from additional participants to compensate for the dropouts. For the fEMG analyses, 10 subjects had to be excluded due to the low EMG activation indicating a loose or broken electrode. Finally, we screened the fEMG data for outliers separately for each cell of the factorial design (see below). No extreme outliers (i.e., more than three interquartile ranges below/above the 25th/75th percentile) were detected. Thus, the sample size was *n* = 38 for behavioral and *n* = 28 for the fEMG analyses.

#### 4.1.2 Stimuli and procedure

Stimuli, design, and procedure were identical with Experiment 2 with the only change that the error feedback was replaced by an uninformative fixation cross.

#### 4.1.3 Data preprocessing

Data preprocessing and outlier identification procedures were the same as in the previous experiments (exclusion of first trial in a block, 7.09% errors, 6.45% post‐error trials, 2.26% responses deviating more than 2.5 *SD*).

### 4.2 Results

#### 4.2.1 Response times

The ANOVA produced a significant main effect of congruency_N_ (congruent: *M* = 482 ms, *SD* = 60 ms; incongruent: *M* = 565 ms, *SD* = 57 ms), *F*(1, 37) = 500.19, *p* ≤ .001, $\eta_{p}^{2}$ = .93. The main effect of congruency_N‐1_ was *ns* , *F*(1, 37) = 3.37, *p* = .075, $\eta_{p}^{2}$ = .08. The interaction between congruency_N_ and congruency_N‐1_ was significant, *F*(1, 37) = 37.51, *p* ≤ .001, $\eta_{p}^{2}$ = .50, indicating conflict adaptation ([*RT_cI_*−*RT_cC_*]–[*RT_iI_*−*RT_iC_*] = 21 ms).

#### 4.2.2 Error rates

ER showed a main effect of congruency_N_, *F*(1, 37) = 77.44, *p* ≤ .001, $\eta_{p}^{2}$ = .68, with more errors in incongruent (*M* = 10.03%, *SD* = 5.96%) compared to congruent (*M* = 4.34%, *SD* = 3.51%) trials. There was no effect of Congruency_N‐1_, *F*(1, 37) = 2.91, *p* = .096, $\eta_{p}^{2}$ = .07. The interaction between both factors was significant, *F*(1, 37) = 6.93, *p* = 012, $\eta_{p}^{2}$ = .16, showing a CSE [(*ER_cI_*−*ER_cC_*)–(*ER_iI_*−*ER_iC_*) = 1.97%; See Table 1].

#### 4.2.3 fEMG

The 2 × 2 × 10 ANOVA of corrugator responses did not show a significant congruency_N_ effect (*F* < 1, *p* = .901; congruent: *M* = 0.01; *SE* = 0.08; 95% CI [−0.15; 0.17]; incongruent: *M* = −0.01; *SE* = 0.08; 95% CI [−0.17; −0.15]; congruency effect: *M_CE_* = −0.02; *SE_CE_* = 0.16; CI [−0.34; 0.30]). The effect of congruency_N‐1_ was also *ns* , *F*(1, 27) = 3.69, *p* = .065, $\eta_{p}^{2}$ = .12. The main effect of time bin, *F*(3, 77) = 5.38, *p* = .002, $\eta_{p}^{2}$ = .17, and the interaction Time Bin × Congruency_N_ were significant, (*F*(6, 167) = 2.99, *p* = .008, $\eta_{p}^{2}$ = .10). The three‐way interaction Congruency_N_ × Congruency_N‐1_ × time bin also reached significance, *F*(6, 154) = 2.41, *p* = .032, $\eta_{p}^{2}$ = .08. Post hoc comparisons did not reveal stable effects across time bins (effects of congruency_N_ were *ns *at any point in time, no linear trend: *F*(1, 27) = 0.02, *p* = .901, $\eta_{p}^{2}$ = .001.), see Figure 1. All other effects were *ns *, all *F*s ≤ 2.89, all *p*s ≥ .1.

We performed Bayesian analyses to test whether the data favor the null hypothesis (absence of a congruency effect) over the alternative hypothesis. The evidence supporting the null model was moderate with BF_01_ = 3.39 (Lee & Wagenmakers, 2013). We also compared the magnitudes of the congruency effects observed in Experiments 2 and 3. No significant difference was observed, *t*(50) = 1.25, *p* = .22, BF_10_ = 1.93. The mean difference of the congruency effects was 0.24, 95% CI: [−0.15; 0.63].

Analyses of the zygomaticus data only showed a significant effect of time bin, *F*(3, 71) = 3.71, *p* = .020, $\eta_{p}^{2}$ = .12, see Figure 1. No other effects reached significance, all *F*s ≤ 1.35, all *p*s ≥ .244. Correlational analyses did not reveal a significant correlation between the fEMG congruency effect and the RT conflict‐adaptation effect, *r*(26) = .28, *p* = .146, and neither for ER, *r*(26) = .05, *p* = .794.

### 4.3 Discussion

The findings of Experiment 3 qualified the findings earlier observed in Experiments 1 and 2. Experiment 3 was identical to Experiment 2 except that we removed the performance feedback. There was no evidence that the facial muscles tracked the putative averseness of conflict, neither in the zygomaticus nor in the corrugator. Bayesian analyses indicated that the model specifying no effect on facial activity (H0) is about three times more likely than the alternative model specifying an effect (H1). At the same time, the experiment produced a conflict‐adaptation effect of similar magnitude as observed in Experiment 2 (see Table 1). An explanation of the null finding due to weak manipulation of conflict is therefore unlikely. We present a possible explanation for the null finding in the next section.

## 5 GENERAL DISCUSSION

The present study used the facial EMG to test the hypothesis that conflict during correct trials in cognitive control tasks is aversive and relates to cognitive control adjustments. Two out of three experiments confirmed the predicted effect, showing increased corrugator activation for conflict relative to no‐conflict trials, both in a flanker task (Experiment 1) and a prime‐probe task (Experiment 2). These findings show for the first time that the aversive response to conflict is reflected in the facial EMG. The marginally significant between‐subject correlation between conflict‐induced corrugator activity and conflict adaptation in Experiment 2 provides the preliminary evidence that this signal is related to subsequent adjustment of cognitive control, which is in line with the predicted functional role of affective signals in the regulation of cognitive control (Dreisbach & Fischer, 2012a, 2015, 2016; Inzlicht et al., 2015; van Steenbergen et al., 2009; van Steenbergen, Band, & Hommel, 2015). In contrast, conflict did not significantly increase corrugator activity in the absence of performance feedback (Experiment 3), and a Bayesian analysis provided moderate evidence in favor of the null hypothesis in that case.

Our findings are consistent with the notion that conflict in cognitive control tasks is aversive, and the hypothesis that affective processes are functionally related to cognitive control (Dreisbach & Fischer, 2015; Inzlicht et al., 2015; van Steenbergen et al., 2015), as supported by several behavioral findings (Dreisbach & Fischer, 2012b; Fritz & Dreisbach, 2013; van Steenbergen et al., 2009). The high temporal resolution of the EMG measure provides additional insights into the temporal dynamics of this putative affective signal. First , the conflict‐driven activation of the corrugator muscle was a response‐locked phenomenon that was not visible in stimulus‐locked analyses. Second , the effect emerged after participants made a response and sustained for the entire 1‐s time window following the response. Our findings are consistent with earlier studies (Lindström et al., 2013; Schacht et al., 2010) that have not observed overall conflict‐induced facial EMG modulation when focusing on pre‐response signals only. However, our findings contrast with traditional measures of neural conflict processes recorded at the scalp which typically precede the response (Cavanagh & Frank, 2014; Larson, Clayson, & Clawson, 2014). Moreover, for conflict trials, we did not observe the typical biphasic response observed for errors, in which the initial aversive facial EMG response is rapidly reversed at the order of half a second later—an effect we have argued to reflect implicit emotion regulation (Dignath et al., 2019). The fact that the sustained post‐response effect correlates with conflict adaption suggests that conflict—even though successfully resolved—has an aversive after‐effect that helps to prepare cognitive control processes in the subsequent trial (cf. Scherbaum, Fischer, Dshemuchadse, & Goschke, 2011).

Given that the control adaptation is an effortful process, one could also argue that the corrugator changes in our study reflect the online recruitment of effort (Botvinick, 2007) rather than the negative valence of the uncertainty associated with stimulus and/or response conflict itself (Mushtaq, Bland, & Schaefer, 2011). This account is consistent with recent frameworks that explain the cognitive control processes in neuroeconomic terms (Shenhav, Botvinick, & Cohen, 2013; Shenhav et al., 2017), and it also fits to previous studies that observed a corrugator increase in conditions that demand effort (Boxtel & Jessurun, 1993; Cacioppo, Petty, & Morris, 1985; de Morree & Marcora, 2010). Because effort is typically aversive (Kool et al., 2010), it is impossible to dissociate effort and negative affect in the present task. However, some recent work has highlighted that people in daily life often seek out cognitive challenges (e.g., solving puzzles or doing video games), suggesting that in some situations effort is actually enjoyable (Inzlicht, Shenhav, & Olivola, 2018). It is an important topic for future studies to measure activity of facial muscles in these situations, which can answer the question of whether corrugator activity reflects affective valence, a general effort signal that is not sensitive to its value, or a combination of both. We have recently developed a method that allows measuring effort‐sensitive cardiac contractility related to task events (Kuipers et al., 2017; Spruit et al., 2018), which provides an additional valuable tool to observe dissociable physiological profiles. In addition to the effect on corrugator, Experiment 2 (but not Experiment 1 and 3) also produced a conflict‐driven reduction in the zygomaticus major. Given the supposed role of this muscle in positive affect, one possible interpretation could be that conflict leads to a reduction of positive affect, which has been suggested before by some behavioral studies (Berger, Fischer, & Dreisbach, 2019; Compton, Huber, Levinson, & Zheutlin, 2012; Damen et al., 2018; Lamers & Roelofs, 2011). However it is difficult to dissociate positive and negative affect in the facial EMG, because facial muscles likely track an integrated, bipolar representation of valence, such that corrugator is activated by negative and deactivated by positive stimuli, whereas zygomaticus shows a reversed—although less reliable—pattern (Lang et al., 1993; Larsen et al., 2003). However, given that the effect was only observed in Experiment 2 (and not in the other two experiments), independent replication of effects in zygomaticus in future studies is warranted.

On a very speculative note, the absence of conflict effects in Experiment 3 might point to the possibility that the presence of performance feedback is an important boundary condition to observe conflict‐driven modulation of the corrugator muscle. It should be noted that errors were not punished in the present research—unlike in other experiments where errors sometimes lead to loss of points or feedback is provided by unpleasant auditory or sensory signals (e.g., Lindström et al., 2013; Yang & Pourtois, 2018). Interestingly, the magnitudes of the conflict‐adaptation effect in Experiment 3 and Experiment 2 were comparable (see the confidence intervals reported in Table 1), suggesting that the conflict adaptation can happen in the absence of aversive signals detectable with EMG. This finding might imply that the aversive response to conflict does not fully mediate the adaptations in cognitive control and that cognitive or lower‐level learning processes independently contribute to control adaptations (Verguts & Notebaert, 2009). Alternatively, besides negative valence, differences in conflict‐induced arousal (van Steenbergen & Band, 2013) can also contribute to the conflict adaptation and it might be speculated that the increased arousal is more important than valence when situations demand endogenous cognitive control such as those not supported by feedback cues. At the same time, however, we cannot rule out the possibility that the effects observed in Experiments 1 and 2 reflect a conditioning effect such that errors were paired with aversive feedback leading to increased aversive signaling after error‐prone incongruent trials. This interpretation would be widely consistent with error‐likelihood accounts of cognitive control that have implied the anterior cingulate cortex in learning prediction to optimize the adaptive recruitment of cognitive control (Brown & Braver, 2005). Our findings thus raise the possibility that the corrugator muscle might index error‐likelihood, in particular in situations where errors are salient. However, it should be also noted that sample size of Experiment 3 was rather small and that a Bayesian test could only provide moderate evidence in favor of the null hypothesis. Most critically, a systematic effect of the presence of feedback was only examined cross‐experimentally by comparison Experiments 2 and 3. Therefore, high‐powered future studies that manipulate the presence of feedback are required to substantiate these speculations.

## 6 CONCLUSIONS

To conclude, our study revealed for the first time the temporal dynamics of the aversive quality of conflict in a cognitive control paradigm. Using the facial EMG, we showed that conflict is associated with increased activation of the corrugator (frowning) muscle after the response and that the size of this effect predicted the size of conflict‐driven control adjustment in the next trial in a confound‐minimized paradigm. This effect was only observed in tasks where participants receive feedback on making errors, suggesting that the facial EMG is particularly sensitive to situations that make errors salient. Our study highlights the potential of using facial EMG measures to test valence‐specific integral emotions in cognitive control tasks and how these might drive adaptations in cognitive control which helps to understand the basic mechanisms underlying adaptive control adaptation (Inzlicht et al., 2015). Applying the method used here might also help to provide insights into the mechanisms underlying disturbed cognitive control, for example in clinical populations (McTeague, Goodkind, & Etkin, 2016).

## AUTHOR CONTRIBUTIONS

All authors were involved in the design of this study. AB and VM collected data. HvS, AB, and VM performed analyses. All authors interpreted the data. AB wrote the first draft and all authors provided critical revisions.

## References

[psyp13524-bib-0001] Achaibou, A. , Pourtois, G. , Schwartz, S. , & Vuilleumier, P. (2008). Simultaneous recording of EEG and facial muscle reactions during spontaneous emotional mimicry. Neuropsychologia, 46(4), 1104–1113. 10.1016/j.neuropsychologia.2007.10.019 18068737

[psyp13524-bib-0002] Berger, A. , Fischer, R. , & Dreisbach, G. (2019). It's more than just conflict: The functional role of congruency in the sequential control adaptation. Acta Psychologica, 197, 64–72. 10.1016/j.actpsy.2019.04.016 31103922

[psyp13524-bib-0003] Botvinick, M. M. (2007). Conflict monitoring and decision making: Reconciling two perspectives on anterior cingulate function. Cognitive, Affective and Behavioral Neuroscience, 7(4), 356–366. 10.3758/CABN.7.4.356 18189009

[psyp13524-bib-0004] Botvinick, M. M. , Braver, T. S. , Barch, D. M. , Carter, C. S. , & Cohen, J. D. (2001). Conflict monitoring and cognitive control. Psychological Review, 108(3), 624–652. 10.1037//0033-295X 11488380

[psyp13524-bib-0005] Braem, S. , Coenen, E. , Bombeke, K. , van Bochove, M. E. , & Notebaert, W. (2015). Open your eyes for prediction errors. Cognitive, Affective and Behavioral Neuroscience, 15(2), 374–380. 10.3758/s13415-014-0333-4 25588818

[psyp13524-bib-0006] Braem, S. , Verguts, T. , Roggeman, C. , & Notebaert, W. (2012). Reward modulates adaptations to conflict. Cognition, 125(2), 324–332. 10.1016/j.cognition.2012.07.015 22892279

[psyp13524-bib-0007] Brouillet, T. , Ferrier, L. P. , Grosselin, A. , & Brouillet, D. (2011). Action compatibility effects are hedonically marked and have incidental consequences on affective judgment. Emotion, 11(5), 1202–1205. 10.1037/a0024742 21875190

[psyp13524-bib-0008] Brown, J. W. , & Braver, T. S. (2005). Learned predictions of error likelihood in the anterior cingulate cortex. Science, 307(5712), 1118–1121. 10.1126/science.1105783 15718473

[psyp13524-bib-0009] Cacioppo, J. T. , Petty, R. E. , & Morris, K. J. (1985). Semantic, evaluative, and self‐referent processing: Memory, cognitive effort, and somatovisceral activity. Psychophysiology, 22(4), 371–384. 10.1111/j.1469-8986.1985.tb01618.x 4023148

[psyp13524-bib-0010] Cannon, P. R. , Hayes, A. E. , & Tipper, S. P. (2010). Sensorimotor fluency influences affect: Evidence from electromyography. Cognition & Emotion, 24(4), 681–691. 10.1080/02699930902927698

[psyp13524-bib-0011] Cavanagh, J. F. , & Frank, M. J. (2014). Frontal theta as a mechanism for cognitive control. Trends in Cognitive Sciences, 18(8), 414–421. 10.1016/j.tics.2014.04.012 24835663 PMC4112145

[psyp13524-bib-0012] Cavanagh, J. F. , Masters, S. E. , Bath, K. , & Frank, M. J. (2014). Conflict acts as an implicit cost in reinforcement learning. Nature Communications, 5, 5394 10.1038/ncomms6394 25367437

[psyp13524-bib-0013] Cavanagh, J. F. , & Shackman, A. J. (2015). Frontal midline theta reflects anxiety and cognitive control: Meta‐analytic evidence. Journal of Physiology‐Paris, 109(1–3), 3–15. 10.1016/j.jphysparis.2014.04.003 PMC4213310 24787485

[psyp13524-bib-0014] Compton, R. J. , Huber, E. , Levinson, A. R. , & Zheutlin, A. (2012). Is “conflict adaptation” driven by conflict? Behavioral and EEG evidence for the underappreciated role of congruent trials. Psychophysiology, 49(5), 583–589. 10.1111/j.1469-8986.2012.01354.x 22332754

[psyp13524-bib-0015] D’Ascenzo, S. , Iani, C. , Guidotti, R. , Laeng, B. , & Rubichi, S. (2016). Practice‐induced and sequential modulations in the Simon task: Evidence from pupil dilation. International Journal of Psychophysiology, 110, 187–193. 10.1016/j.ijpsycho.2016.08.002 27503609

[psyp13524-bib-0016] Damen, T. G. E. , Strick, M. , Taris, T. W. , & Aarts, H. (2018). When conflict influences liking: The case of the stroop task. PLoS ONE, 13(7), 1–23. 10.1371/journal.pone.0199700 PMC6040704 29995919

[psyp13524-bib-0017] Darwin, C. (1872 [2009]). The expression of the emotions in man and animals. New York, NY: Oxford University Press.

[psyp13524-bib-0018] Davelaar, E. J. , & Stevens, J. (2009). Sequential dependencies in the Eriksen flanker task: A direct comparison of two competing accounts. Psychonomic Bulletin and Review, 16(1), 121–126. 10.3758/PBR.16.1.121 19145021

[psyp13524-bib-0019] de Morree, H. M. , & Marcora, S. M. (2010). The face of effort: Frowning muscle activity reflects effort during a physical task. Biological Psychology, 85(3), 377–382. 10.1016/j.biopsycho.2010.08.009 20832447

[psyp13524-bib-0020] de Ridder, D. T. D. , Lensvelt‐Mulders, G. , Finkenauer, C. , Stok, F. M. , & Baumeister, R. F. (2012). Taking stock of self‐control: A meta‐analysis of how trait self‐control relates to a wide range of behaviors. Personality and Social Psychology Review, 16(1), 76–99. 10.1177/1088868311418749 21878607

[psyp13524-bib-0021] Diede, N. T. , & Bugg, J. M. (2017). Cognitive effort is modulated outside of the explicit awareness of conflict frequency: Evidence from pupillometry. Journal of Experimental Psychology: Learning Memory and Cognition, 43(5), 824–835. 10.1037/xlm0000349 PMC5411282 28068124

[psyp13524-bib-0022] Dignath, D. , Berger, A. , Spruit, I. M. , & van Steenbergen, H. (2019). Temporal dynamics of error‐related corrugator supercilii and zygomaticus major activity: Evidence for implicit emotion regulation following errors. International Journal of Psychophysiology, 146, 208–216. 10.1016/j.ijpsycho.2019.10.003 31648024

[psyp13524-bib-0023] Dignath, D. , & Eder, A. B. (2015). Stimulus conflict triggers behavioral avoidance. Cognitive, Affective, & Behavioral Neuroscience, 15(4), 822–836. 10.3758/s13415-015-0355-6 25931151

[psyp13524-bib-0025] Dignath, D. , Eder, A. B. , Steinhauser, M. , & Kiesel, A. . (2020). Conflict monitoring and the affectivesignaling hypothesis—An integrative review. Psychonomic Bulletin & Review, 1–24. 10.3758/s13423-019-01668-9 31898269

[psyp13524-bib-0024] Dignath, D. , Janczyk, M. , & Eder, A. B. (2017). Phasic valence and arousal do not influence post‐conflict adjustments in the Simon task. Acta Psychologica, 174, 31–39. 10.1016/j.actpsy.2017.01.004 28135636

[psyp13524-bib-0026] Dimberg, U. (1990). Facial electromyographic reactions and autonomic activity to auditory stimuli. Biological Psychology, 31(2), 137–147. 10.1016/0301-0511(90)90013-M 2103748

[psyp13524-bib-0027] Dimberg, U. , & Karlsson, B. (1997). Facial reactions to different emotionally relevant stimuli. Scandinavian Journal of Psychology, 38(4), 297–303. 10.1111/1467-9450.00039

[psyp13524-bib-0028] Dreisbach, G. , & Fischer, R. (2012a). The role of affect and reward in the conflict‐triggered adjustment of cognitive control. Frontiers in Human Neuroscience, 6, 342 10.3389/fnhum.2012.00342 23293597 PMC3533233

[psyp13524-bib-0029] Dreisbach, G. , & Fischer, R. (2012b). Conflicts as aversive signals. Brain and Cognition, 78(2), 94–98. 10.1016/j.bandc.2011.12.003 22218295

[psyp13524-bib-0030] Dreisbach, G. , & Fischer, R. (2015). Conflicts as aversive signals for control adaptation. Current Directions in Psychological Science, 24(4), 255–260. 10.1177/0963721415569569

[psyp13524-bib-0031] Dreisbach, G. , & Fischer, R. (2016). Conflicts as aversive signals: Motivation for control adaptation in the service of affect regulation In Braver T. S. (Ed.), Motivation and cognitive control (pp. 188–210). New York, NY: Psychology Press.

[psyp13524-bib-0032] Duthoo, W. , Abrahamse, E. L. , Braem, S. , Boehler, C. N. , & Notebaert, W. (2014). The heterogeneous world of congruency sequence effects: An update. Frontiers in Psychology, 5, 1001 10.3389/fpsyg.2014.01001 25250005 PMC4158803

[psyp13524-bib-0034] Egner, T. (2007). Congruency sequence effects and cognitive control. Cognitive, Affective, & Behavioral Neuroscience, 7(4), 380–390. 10.3758/CABN.7.4.380 18189011

[psyp13524-bib-0036] Elkins‐Brown, N. , Saunders, B. , He, F. , & Inzlicht, M. (2017). Stability and reliability of error‐related electromyography over the corrugator supercilii with increasing trials. Psychophysiology, 54, 1559–1573. 10.1111/psyp.12556 28621433

[psyp13524-bib-0037] Elkins‐Brown, N. , Saunders, B. , & Inzlicht, M. (2016). Error‐related electromyographic activity over the corrugator supercilii is associated with neural performance monitoring. Psychophysiology, 53(2), 159–170. 10.1111/psyp.12556 26470645

[psyp13524-bib-0038] Forster, M. , Leder, H. , & Ansorge, U. (2016). Exploring the subjective feeling of fluency. Experimental Psychology, 63(1), 45–58. 10.1027/1618-3169/a000311 27025534

[psyp13524-bib-0039] Fritz, J. , & Dreisbach, G. (2013). Conflicts as aversive signals: Conflict priming increases negative judgments for neutral stimuli. Cognitive, Affective and Behavioral Neuroscience, 13(2), 311–317. 10.3758/s13415-012-0147-1 23307475

[psyp13524-bib-0040] Fritz, J. , & Dreisbach, G. (2015). The time course of the aversive conflict signal. Experimental Psychology, 62, 30–39. 10.1027/1618-3169/a000271 25270558

[psyp13524-bib-0041] Fröber, K. , Stürmer, B. , Frömer, R. , & Dreisbach, G. (2017, April). The role of affective evaluation in conflict adaptation: An LRP study. Brain and Cognition, 116, 9–16. 10.1016/j.bandc.2017.05.003 28570905

[psyp13524-bib-0042] Gerger, G. , Leder, H. , Tinio, P. P. , & Schacht, A. (2011). Faces versus patterns: Exploring aesthetic reactions using facial EMG. Psychology of Aesthetics, Creativity, and the Arts, 5(3), 241–250. 10.1037/a0024154

[psyp13524-bib-0043] Gratton, G. , Coles, M. G. , & Donchin, E. (1992). Optimizing the use of information: Strategic control of activation of responses. Journal of Experimental Psychology: General, 121(4), 480–506. 10.1037/0096-3445.121.4.480 1431740

[psyp13524-bib-0044] Gronau, Q. F. , Ly, A. , & Wagenmakers, E. J. (2019). Informed Bayesian *t*‐tests. The American Statistician, 73 10.1080/00031305.2018.1562983

[psyp13524-bib-0045] Hommel, B. (2004). Event files: Feature binding in and across perception and action. Trends in Cognitive Sciences, 8(11), 494–500. 10.1016/j.tics.2004.08.007 15491903

[psyp13524-bib-0046] Inzlicht, M. , Bartholow, B. D. , & Hirsh, J. B. (2015). Emotional foundations of cognitive control. Trends in Cognitive Sciences, 19(3), 126–132. 10.1016/j.tics.2015.01.004 25659515 PMC4348332

[psyp13524-bib-0047] Inzlicht, M. , Shenhav, A. , & Olivola, C. Y. (2018). The effort paradox: Effort is both costly and valued. Trends in Cognitive Sciences, 22(4), 337–349. 10.1016/j.tics.2018.01.007 29477776 PMC6172040

[psyp13524-bib-0048] Kerns, J. G. , Cohen, J. D. , MacDonald, A. W. , Cho, R. Y. , Stenger, V. A. , & Carter, C. S. (2004). Anterior cingulate conflict monitoring and adjustments in control. Science, 303(5660), 1023–1026. 10.1126/science.1089910 14963333

[psyp13524-bib-0049] Kobayashi, N. , Yoshino, A. , Takahashi, Y. , & Nomura, S. (2007). Autonomic arousal in cognitive conflict resolution. Autonomic Neuroscience, 132(1–2), 70–75. 10.1016/j.autneu.2006.09.004 17067858

[psyp13524-bib-0050] Kool, W. , McGuire, J. T. , Rosen, Z. B. , & Botvinick, M. M. (2010). Decision making and the avoidance of cognitive demand. Journal of Experimental Psychology: General, 139(4), 665–682. 10.1037/a0020198 20853993 PMC2970648

[psyp13524-bib-0051] Kragel, P. A. , Kano, M. , Van Oudenhove, L. , Ly, H. G. , Dupont, P. , Rubio, A. , … Wager, T. D. (2018). Generalizable representations of pain, cognitive control, and negative emotion in medial frontal cortex. Nature Neuroscience, 21(2), 283–289. 10.1038/s41593-017-0051-7 29292378 PMC5801068

[psyp13524-bib-0052] Kuhbandner, C. , & Zehetleitner, M. (2011). Dissociable effects of valence and arousal in adaptive executive control. PLoS ONE, 6(12), e29287 10.1371/journal.pone.0029287 22216233 PMC3244450

[psyp13524-bib-0053] Kuipers, M. , Richter, M. , Scheepers, D. , Immink, M. A. , Sjak-Shie, E. , & van Steenbergen, H. (2017). How effortful is cognitive control? Insights from a novel method measuring single-trial evoked betaadrenergic cardiac reactivity. International Journal of Psychophysiology, 119, 87–92. 10.1016/j.ijpsycho.2016.10.007 27737782

[psyp13524-bib-0054] Lamers, M. J. M. , & Roelofs, A. (2011). Attentional control adjustments in Eriksen and stroop task performance can be independent of response conflict. Quarterly Journal of Experimental Psychology, 64(6), 1056–1081. 10.1080/17470218.2010.523792 21113864

[psyp13524-bib-0055] Lang, P. J. , Greenwald, M. K. , Bradley, M. M. , & Hamm, A. O. (1993). Looking at pictures: Affective, facial, visceral, and behavioral reactions. Psychophysiology, 30(3), 261–273. 10.1111/j.1469-8986.1993.tb03352.x 8497555

[psyp13524-bib-0056] Larson, M. J. , Clayson, P. E. , & Clawson, A. (2014). Making sense of all the conflict: A theoretical review and critique of conflict-related ERPs. International Journal of Psychophysiology, 93(3), 283–297. 10.1016/j.ijpsycho.2014.06.007 24950132

[psyp13524-bib-0057] Larsen, J. T. , Norris, C. J. , & Cacioppo, J. T. (2003). Effects of positive and negative affect on electromyographic activity over zygomaticus major and corrugator supercilii. Psychophysiology, 40, 776–785. 10.1111/1469-8986.00078 14696731

[psyp13524-bib-0058] Lee, M. D. , & Wagenmakers, E. J. (2013). Bayesian data analysis for cognitive science: A practical course. New York, NY: Cambridge University Press.

[psyp13524-bib-0059] Lindström, B. R. , Mattsson‐Mårn, I. B. , Golkar, A. , & Olsson, A. (2013). In your face: Risk of punishment enhances cognitive control and error‐related activity in the corrugator supercilii muscle. PLoS ONE, 8(6), e65692 10.1371/journal.pone.0065692 23840356 PMC3694071

[psyp13524-bib-0060] Mayr, U. , Awh, E. , & Laurey, P. (2003). Conflict adaptation effects in the absence of executive control. Nature Neuroscience, 6(5), 450–452. 10.1038/nn1051 12704394

[psyp13524-bib-0061] McTeague, L. M. , Goodkind, M. S. , & Etkin, A. (2016). Transdiagnostic impairment of cognitive control in mental illness. Journal of Psychiatric Research, 83, 37–46. 10.1016/j.jpsychires.2016.08.001 27552532 PMC5107153

[psyp13524-bib-0062] Morsella, E. , Gray, J. R. , Krieger, S. C. , & Bargh, J. A. (2009). The essence of conscious conflict: Subjective effects of sustaining incompatible intentions. Emotion, 9(5), 717–728. 10.1037/a0017121 19803593 PMC2762124

[psyp13524-bib-0063] Moser, J. , Moran, T. , Schroder, H. , Donnellan, B. , & Yeung, N. (2013). On the relationship between anxiety and error monitoring: A meta‐analysis and conceptual framework. Frontiers in Human Neuroscience, 7, 466 10.3389/fnhum.2013.00466 23966928 PMC3744033

[psyp13524-bib-0064] Murphy, P. R. , Van Moort, M. L. , & Nieuwenhuis, S. (2016). The pupillary orienting response predicts adaptive behavioral adjustment after errors. PLoS ONE, 11(3), 1–11. 10.1371/journal.pone.0151763 PMC4807057 27010472

[psyp13524-bib-0065] Mushtaq, F. , Bland, A. R. , & Schaefer, A. (2011). Uncertainty and cognitive control. Frontiers in Psychology, 2, 249 10.3389/fpsyg.2011.00249 22007181 PMC3184613

[psyp13524-bib-0066] Nieuwenhuis, S. , Stins, J. F. , Posthuma, D. , Polderman, T. J. C. , Boomsma, D. I. , & De Geus, E. J. (2006). Accounting for sequential trial effects in the flanker task: Conflict adaptation or associative priming? Memory and Cognition, 34(6), 1260–1272. 10.3758/BF03193270 17225507

[psyp13524-bib-0067] Oster, H. (1978). Facial expression and affect development In Lewis M. & Rosenblum L. A. (Eds.), The development of affect (pp. 43–75). New York, NY: Plenum.

[psyp13524-bib-0068] Pan, F. , Shi, L. , Lu, Q. , Wu, X. , Xue, S. , & Li, Q. (2016). The negative priming effect in cognitive conflict processing. Neuroscience Letters, 628, 35–39. 10.1016/j.neulet.2016.05.062 27268038

[psyp13524-bib-0069] Quintana, D. S. , & Williams, D. R. (2018). Bayesian alternatives for common null‐hypothesis significance tests in psychiatry: A non‐technical guide using JASP. BMC Psychiatry, 18, 178 10.1186/s12888-018-1761-4 29879931 PMC5991426

[psyp13524-bib-0070] Regenberg, N. F. , Häfner, M. , & Semin, G. R. (2012). The groove move: Action affordances produce fluency and positive affect. Experimental Psychology, 59(1), 30–37. 10.1027/1618-3169/a000122 21768065

[psyp13524-bib-0071] Riesel, A. (2019). The erring brain: Error‐related negativity as an endophenotype for OCD—A review and meta‐analysis. Psychophysiology, 56(4), e13348 10.1111/psyp.13348 30838682

[psyp13524-bib-0072] Rinn, W. E. (1984). The neuropsychology of facial expression: A review of the neurological and psychological mechanisms for producing facial expressions. Psychological Bulletin, 95(1), 52–77. 10.1037/0033-2909.95.1.52 6242437

[psyp13524-bib-0073] Schacht, A. K. , Dimigen, O. , & Sommer, W. (2010). Emotions in cognitive conflicts are not aversive but are task specific. Cognitive, Affective and Behavioral Neuroscience, 10(3), 349–356. 10.3758/CABN.10.3.349 20805536

[psyp13524-bib-0074] Scherbaum, S. , Fischer, R. , Dshemuchadse, M. , & Goschke, T. (2011). The dynamics of cognitive control: Evidence for within‐trial conflict adaptation from frequency‐tagged EEG. Psychophysiology, 48(5), 591–600. 10.1111/j.1469-8986.2010.01137.x 21044093

[psyp13524-bib-0075] Schmidt, J. R. (2013). Questioning conflict adaptation: Proportion congruent and Gratton effects reconsidered. Psychonomic Bulletin & Review, 20(4), 615–630. 10.3758/s13423-012-0373-0 23325703

[psyp13524-bib-0076] Schmidt, J. R. , & Weissman, D. H. (2014). Congruency sequence effects without feature integration or contingency learning confounds. PLoS ONE, 9(7), e102337 10.1371/journal.pone.0102337 25019526 PMC4096924

[psyp13524-bib-0077] Schouppe, N. , Braem, S. , De Houwer, J. , Silvetti, M. , Verguts, T. , Ridderinkhof, K. R. , & Notebaert, W. (2015). No pain, no gain: The affective valence of congruency conditions changes following a successful response. Cognitive, Affective and Behavioral Neuroscience, 15(1), 251–261. 10.3758/s13415-014-0318-3 25183556

[psyp13524-bib-0078] Schuch, S. , & Koch, I. (2015). Mood states influence cognitive control: The case of conflict adaptation. Psychological Research Psychologische Forschung, 79(5), 759–772. 10.1007/s00426-014-0602-4 25100233

[psyp13524-bib-0079] Schuch, S. , Zweerings, J. , Hirsch, P. , & Koch, I. (2017). Conflict adaptation in positive and negative mood: Applying a success‐failure manipulation. Acta Psychologica, 176, 11–22. 10.1016/j.actpsy.2017.03.005 28342397

[psyp13524-bib-0080] Shackman, A. J. , Salomons, T. V. , Slagter, H. A. , Fox, A. S. , Winter, J. J. , & Davidson, R. J. (2011). The integration of negative affect, pain and cognitive control in the cingulate cortex. Nature Reviews Neuroscience, 12(3), 154–167. 10.1038/nrn2994 21331082 PMC3044650

[psyp13524-bib-0081] Shenhav, A. , Botvinick, M. M. , & Cohen, J. D. (2013). The expected value of control: An integrative theory of anterior cingulate cortex function. Neuron, 79(2), 217–240. 10.1016/j.neuron.2013.07.007 23889930 PMC3767969

[psyp13524-bib-0082] Shenhav, A. , Musslick, S. , Lieder, F. , Kool, W. , Griffiths, T. L. , Cohen, J. D. , & Botvinick, M. M. (2017). Toward a rational and mechanistic account of mental effort. Annual Review of Neuroscience, 40, 99–124. 10.1016/j.neuron.2013.07.007 28375769

[psyp13524-bib-0083] Spapé, M. M. , & Ravaja, N. (2016, April). Not my problem: Vicarious conflict adaptation with human and virtual co‐actors. Frontiers in Psychology, 7, 1–13. 10.3389/fpsyg.2016.00606 27199839 PMC4848756

[psyp13524-bib-0084] Spruit, I. M. , Wilderjans, T. F. , & van Steenbergen, H. (2018). Heart work after errors: Behavioral adjustment following error commission involves cardiac effort. Cognitive, Affective and Behavioral Neuroscience, 18(2), 375–388. 10.3758/s13415-018-0576-6 PMC5889424 29464553

[psyp13524-bib-0085] Topolinski, S. , Likowski, K. U. , Weyers, P. , & Strack, F. (2009). The face of fluency: Semantic coherence automatically elicits a specific pattern of facial muscle reactions. Cognition and Emotion, 23(2), 260–271. 10.1080/02699930801994112

[psyp13524-bib-0086] Van Boxtel, A. , & Jessurun, M. (1993). Amplitude and bilateral coherency of facial and jaw‐elevator EMG activity as an index of effort during a two‐choice serial reaction task. Psychophysiology, 30(6), 589–604. 10.1111/j.1469-8986.1993.tb02085.x 8248451

[psyp13524-bib-0087] van Steenbergen, H. (2015). Affective modulation of cognitive control: A biobehavioral perspective In Gendolla G., Tops M., & Koole S. (Eds.), Handbook of biobehavioral approaches to self‐regulation (pp. 89–107). New York, NY: Springer.

[psyp13524-bib-0088] van Steenbergen, H. , & Band, G. P. H. (2013, May). Pupil dilation in the Simon task as a marker of conflict processing. Frontiers in Human Neuroscience, 7, 1–11. 10.3389/fnhum.2013.00215 23754997 PMC3665936

[psyp13524-bib-0089] van Steenbergen, H. , Band, G. P. H. , & Hommel, B. (2009). Reward counteracts conflict adaptation: Evidence for a role of affect in executive control. Psychological Science, 20(12), 1473–1477. 10.1111/j.1467-9280.2009.02470.x 19906127

[psyp13524-bib-0090] van Steenbergen, H. , Band, G. P. H. , & Hommel, B. (2010). In the mood for adaptation: How affect regulates conflict‐driven control. Psychological Science, 21(11), 1629–1634. 10.1177/0956797610385951 20943936

[psyp13524-bib-0091] van Steenbergen, H. , Band, G. P. H. , & Hommel, B. (2015, July). Does conflict help or hurt cognitive control? Initial evidence for an inverted U‐shape relationship between perceived task difficulty and conflict adaptation. Frontiers in Psychology, 6, 1–17. 10.3389/fpsyg.2015.00974 26217287 PMC4498021

[psyp13524-bib-0092] Verguts, T. , & Notebaert, W. (2009). Adaptation by binding: A learning account of cognitive control. Trends in Cognitive Sciences, 13(6), 252–257. 10.1016/j.tics.2009.02.007 19428288

[psyp13524-bib-0093] Wessel, J. R. , Danielmeier, C. , & Ullsperger, M. (2011). Error awareness revisited : Accumulation of multimodal evidence from central and autonomic nervous systems. Journal of Cognitive Neuroscience, 23(10), 3021–3036. 10.1162/jocn.2011.21635 21268673

[psyp13524-bib-0094] Wessel, J. R. , O'Doherty, J. P. , Berkebile, M. M. , Linderman, D. , & Aron, A. R. (2014). Stimulus devaluation induced by stopping action. Journal of Experimental Psychology: General, 143(6), 2316–2329. 10.1037/xge0000022 25313953 PMC4244281

[psyp13524-bib-0095] Winkielman, P. , & Cacioppo, J. T. (2001). Mind at ease puts a smile on the face: Psychophysiological evidence that processing facilitation elicits positive affect. Journal of Personality and Social Psychology, 81(6), 989–1000. 10.1037//0022-3514.81.6.989 11761320

[psyp13524-bib-0096] Yamaguchi, M. , & Nishimura, A. (2019). Modulating proactive cognitive control by reward: Differential anticipatory effects of performance‐contingent and non‐contingent rewards. Psychological Research Psychologische Forschung, 83(2), 258–274. 10.1007/s00426-018-1027-2 29855699 PMC6433802

[psyp13524-bib-0097] Yang, Q. , & Pourtois, G. (2018). Conflict‐driven adaptive control is enhanced by integral negative emotion on a short time scale. Cognition and Emotion, 32(8), 1637–1653. 10.1080/02699931.2018.1434132 29400596

