# Supplementary material for: The face of control: Corrugator supercilii tracks aversive conflict signals in the service of adaptive cognitive control
Source: Psychophysiology. 2020 Jan 13;57(4):e13524. doi: 10.1111/psyp.13524 (PMC7079141; doi:10.1111/psyp.13524)
Supplement: Supplementary file 1 [file PSYP-57-e13524-s002.docx]

**Data and analysis scripts are available online:**

<https://osf.io/ukj7w/>
