# Supplementary material for: The face of control: Corrugator supercilii tracks aversive conflict signals in the service of adaptive cognitive control
Source: Psychophysiology. 2020 Jan 13;57(4):e13524. doi: 10.1111/psyp.13524 (PMC7079141; doi:10.1111/psyp.13524)
Supplement: Supplementary file 2 [file PSYP-57-e13524-s001.docx]

# Supplementary Material

## Methods

Data preprocessing and outlier identification procedures were the same as described in the main text up until the point of z-transformation. The results presented in this section are based on unstandardized data. The unstandardized fEMG data were checked for outliers separately for each cell of the factorial design (see below). In comparison to the data reported in the main text, more participants were identified as outliers (i.e., more than 3 interquartile ranges below/above the 25^th^/75^th^ percentile in at least one cell) in the unstandardized data sets: Four subjects were excluded in Experiment 1 (*n* = 42), four outliers were excluded in Experiment 2 (*n* = 21) and six outliers were excluded in Experiment 3 (*n* = 22).

## Results

Unstandardized fEMG data for all three experiments are presented in Figure S1.

### Experiment 1

The 2 × 2 × 10 ANOVA of corrugator activation did not show a significant effect for Congruency_N_, *F*(1,45) = 2.91, *p* = .096. No other effects reached significance either, all *F*s ≤ 0.53, all *p*s ≥ .47.

An ANOVA for zygomaticus data revealed a significant effect of time bin, *F*(4,169) = 4.73, *p* ≤ .001, $\eta_{p}^{2}$ = .11. Activation increased over time following a linear trend, *F*(1,40) = 9.75, *p* ≤=.003, $\eta_{p}^{2}$ = .20. No other effects were significant, all *F*s ≤ 3.12, all *p*s ≥ .06. Results were therefore unchanged to the analysis of unstandardized data.

### Experiment 2

The 2 × 2 × 10 ANOVA of corrugator activation revealed a main effect of Congruency_N_, *F*(1,20) = 6.04, *p* = .023, $\eta_{p}^{2}$ = .23, indicating stronger activation in incongruent trials (*M* = -0.05; *SE* = 0.10; 95% CI [-0.27; 0.161]) than in congruent trials (*M* = -0.13; *SE* = 0.11; 95% CI [-0.37; 0.11]; *M_CE_* = 0.07; *SE* = 0.03; 95% CI [0.01; 0.14]). No other effects reached significance, all *F*s ≤ 2.39, all *p*s ≥ .09.

Analyses of the zygomaticus activation showed a marginally significant main effect of Congruency_N_, *F* = 4.14, *p* = .06, $\eta_{p}^{2}$ = .17 (*M_CE_* = -0.025; *SE* = 0.012; 95% CI [-0.051; 0.001]. No other effects were significant, all *F*s ≤ 1.63, all *p*s ≥ .11.

### Experiment 3

The 2 × 2 × 10 ANOVA of corrugator responses did not show a significant Congruency_N_ effect (*F* < 1, *p* = .59; congruent: *M* = -0.09; *SE* = 0.10; 95% CI [-0.30; 0.11]; incongruent: *M* = -0.06; *SE* = 0.13; 95% CI [-0.32; 0.20]). The effect of Congruency_N-1_ was also not significant, *F* < 1.39, *p* = .25. The main effect of time bin was significant, *F*(2,43) = 6.26, *p* = .004, $\eta_{p}^{2}$ = .23, but the interaction time bin × Congruency_N_ was not, *p* = .058. Neither was the three-way interaction Congruency_N_ × Congruency_N-1_ × time bin, *p* = .219.

Analyses of the zygomaticus activation showed no significant effects but the effect of time bin, *F (3, 53)* = 3.73, *p* = .02, $\eta_{p}^{2}$ = .15.


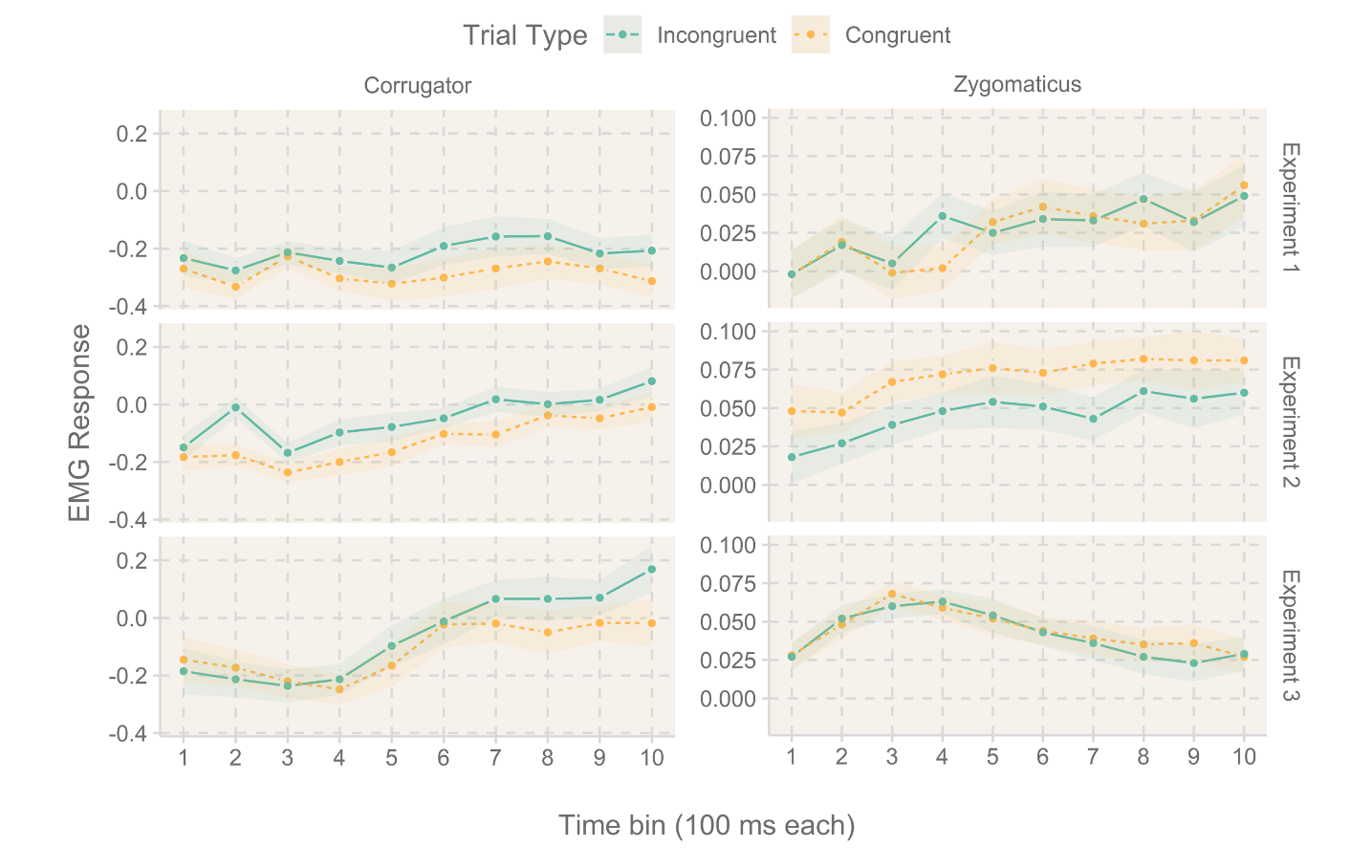


*Figure S1.* Grand averages of unstandardized EMG activation (RMS) in mV of the corrugator and zygomaticus muscles as a function of each time bin and congruency in the three experiments. Shaded areas represent within-subject standard errors.
